# The Link Between Physical Activity, Nutrition, and Health: A Cross-Sectional Study with Multivariate Analysis in a Young and Predominantly Female Spanish Sample

**DOI:** 10.3390/nu17091486

**Published:** 2025-04-28

**Authors:** Elena Sandri, Michela Capoferri, Gaia Luciani, Michela Piredda

**Affiliations:** 1 Faculty of Medicine and Health Sciences, Catholic University of Valencia San Vicente Mártir, C/Quevedo 2, 46001 Valencia, Spain; 2 Department of Animal Production and Health, Veterinary Public Health, and Food Science and Technology, Institute of Biomedical Sciences, Faculty of Veterinary Medicine, Cardenal Herrera-CEU University, Calle Santiago Ramón y Cajal 20, 46115 Alfara del Patriarca, Spain; michela.capoferricapoferri@alumnos.uchceu.es; 3 Department of Biomedicine and Prevention, Tor Vergata University, Via Montpellier 1, 00133 Rome, Italy; gaia.luciani.gl@gmail.com; 4 Research Unit Nursing Science, Department of Medicine and Surgery, Campus Bio-Medico di Roma University, Via Alvaro del Portillo 21, 00128 Rome, Italy

**Keywords:** lifestyle habits, healthy habits, sport, physical activity, socio-demographic factors

## Abstract

Background: Physical activity and nutrition play a crucial role in maintaining overall health and well-being. This cross-sectional study analyzes the relationship between physical activity habits and dietary patterns in the Spanish population. Methods: Data were collected through validated questionnaires: the Nutritional and Social Healthy Habits scale (NutSo-HH) for nutritional and lifestyle habits and the Global Physical Activity Questionnaire for physical activity distributed via social media from June to November 2024. Results: The sample included 1534 respondents (67% female), aged 18–65+ years, with diverse educational, income, and living conditions. Socio-demographic, nutritional, lifestyle, and physical activity data were analyzed using non-parametric statistical tests and Principal Component Analysis. On average, participants engaged in 96.7 min/week of high-intensity physical activity and 118 min/week) of moderate-intensity physical activity. While 75% met the WHO recommendations for moderate activity, only 40% reached optimal levels of high-intensity activity. Individuals engaging in higher levels of physical activity, particularly high-intensity exercise, reported better self-perceived health, lower obesophobia, and better body image. Additionally, a positive correlation was found between higher physical activity levels and the consumption of fruits, vegetables, legumes, and fish, while individuals with lower physical activity levels demonstrated a higher intake of ultra-processed and fried foods. Conclusions: The Principal Component Analysis revealed a bidirectional relationship between the alignment of healthy dietary habits and increased physical activity. These findings highlight the importance of promoting both physical activity and balanced nutrition to enhance overall health and lifestyle quality.

## 1. Introduction

Health outcomes are strongly influenced by the relationship between physical activity (PA) and nutrition, both of which contribute to metabolic efficiency, disease prevention, and overall well-being [1]. While many studies examine these factors individually, emerging evidence suggests their combined benefits often surpass those observed separately [2,3,4]. This integrated perspective is essential not only for optimizing body composition and musculoskeletal health but also for enhancing cognitive and physical performance across the lifespan [2]. Regular PA is associated with a range of health benefits, including the prevention and management of chronic diseases such as cardiovascular disease, type 2 diabetes, certain cancers, hypertension, depression, and osteoporosis [5]. Furthermore, regular PA improves physiological fitness and supports functional independence, enhancing overall quality of life [6]. Understanding the combined role of PA and nutrition is vital for developing effective health strategies that support long-term well-being and mitigate the rise of metabolic disorders, which are becoming increasingly prevalent worldwide.

Despite these benefits, physical inactivity remains a global concern [7]. In 2022, 31% of adults worldwide—approximately 1.8 billion people—did not meet the recommended PA levels, and this trend is expected to worsen, with a projected 35% increase in physical inactivity by 2030 [2]. Individuals who engage in moderate or vigorous PA have a significantly lower risk of cardiovascular mortality, regardless of metabolic risk factors. In contrast, physical inactivity and sedentary behaviors increase the risk of type 2 diabetes, regardless of age, gender, or body mass index [8]. Recent research points to excessive screen time and limited recreational spaces in urban environments as major contributors to sedentary behavior, especially among adolescents. These factors have significant negative effects on both physical and mental health [9].

Promoting PA from an early age is a key public health strategy, as childhood obesity strongly predicts its persistence into adulthood, with approximately 70% of obese children remaining obese as adults. Therefore, fostering early engagement in PA, along with the promotion of healthy eating habits, not only improves immediate health outcomes but also contributes to long-term obesity prevention, reducing the burden of metabolic disorders on healthcare systems [10].

Similarly, unhealthy dietary patterns, characterized by excessive consumption of ultra-processed foods, refined sugars, and saturated fats, are strongly associated with increased adiposity, insulin resistance, and systemic inflammation, all of which contribute to the global burden of chronic diseases [11]. Moreover, the National Weight Control Registry highlights that long-term weight loss maintenance is achievable through a combination of regular PA, a low-calorie, low-fat diet, and consistent eating habits, demonstrating the importance of integrating both exercise and dietary strategies for effective weight management [12]. Understanding how these factors interact in real-life settings is crucial for designing targeted interventions to improve population health outcomes.

Although there are studies on the relationship between physical activity and nutrition in Spain, many focus on specific demographic groups or use traditional methodological approaches. For example, Tárraga López et al. [13] investigate the relationship between physical activity and Mediterranean diet adherence among Spanish university students, focusing on the level and type of physical activity. Similarly, Redondo Del Río et al. [14] compare dietary intake and adherence to the Mediterranean diet between athletes and non-athletes, and Guillén Alcolea et al. [15] explore the association between obesity, physical activity, Mediterranean diet adherence, and body dissatisfaction in individuals aged 16–50 years in the Region of Murcia.

This cross-sectional study, conducted on a large and diverse Spanish sample, aimed to examine the relationship between PA habits and dietary patterns, the influence of socio-demographic factors—such as age, gender, education, and income—on these behaviors, and to assess how PA and diet correlates with self-reported health outcomes, including perceived health status and body image. We hypothesize that higher PA levels correspond to healthier dietary choices, whereas lower PA levels are linked to greater consumption of ultra-processed and fried foods. Additionally, individuals who engage in more intense PA are likely to report better self-perceived health and a more positive body image.

## 2. Materials and Methods

### 2.1. Study Design and Sampling

This study utilized a cross-sectional design with a quantitative approach. A non-probabilistic snowball technique [16] was employed, in which participants were initially recruited through randomly selected individuals on social networks. These initial participants then invited others who met the predefined inclusion criteria, generating a continuous recruitment chain, often referred to as a “snowball effect”. To ensure a diverse and representative sample, individuals from various age groups and geographic regions across Spain were included in this study.

### 2.2. Inclusion and Exclusion Criteria

The inclusion criteria for this study required participants to be 18 years or older, of Spanish nationality, and residing in Spain. The following exclusion criteria were to prevent potential biases: individuals diagnosed with chronic conditions that could significantly influence their dietary patterns or those experiencing temporary circumstances that disrupted their usual diet, such as hospitalization or imprisonment.

### 2.3. Ethical Considerations

This study adhered to the ethical guidelines of the Declaration of Helsinki [17] and received approval from the Research Ethics Committee of the Catholic University of Valencia (approval code UCV/2023-2024/192, 28 May 2024). Data collected were pseudo-anonymized, and no personally identifiable information was recorded. Potential participants were informed about this study’s aims, procedures, anonymous data collection, and aggregated data analysis. They were assured that participation was voluntary and optional. Before providing consent, participants were invited to read the privacy and data processing information. Only those who explicitly agreed to participate by selecting a consent checkbox were allowed to proceed with the survey.

### 2.4. Data Collection

The questionnaire was hosted on Google Forms and distributed using a non-probabilistic snowball sampling approach, with Instagram serving as the primary platform. An Instagram account, @elretonutricional, was created specifically for this study, enabling professionals, influencers, and supporters to assist in distributing the questionnaire. Additionally, researchers shared the survey through LinkedIn, Twitter, WhatsApp, and Facebook. Data collection was conducted between June 2024 and November 2024.

### 2.5. Measurements

This study employed two validated questionnaires:

1. The Nutritional and Social Healthy Habits scale (NutSo-HH) [18] collected detailed information on nutritional, social, and lifestyle habits, recording choices or frequency of consumption. The instrument has undergone rigorous psychometric testing and is validated as a reliable tool.
2. The Global Physical Activity Questionnaire (GPAQ) is a widely translated and validated instrument [19] that assessed respondents’ physical activity habits, differentiating between moderate- and high-intensity physical activity in both professional and leisure settings.

Nutritional variables were categorized using a Likert scale ranging from 1 to 4, following criteria established in previous articles [20,21]. A score of 1 indicates no or low frequency, while a score of 4 corresponds to maximum frequency. The survey also explored variables indicative of potential eating disorder symptoms, such as concerns about gaining weight or feeling overweight, lack of control over food intake, feelings of shame after eating, and preoccupation with body shape. These variables were assessed using a 6-point Likert scale (ranging from 1 = never to 6 = always). Finally, a detailed categorization of health and lifestyle variables, except for body mass index (BMI), which was recorded as a continuous numerical value, is provided in Table 1.

#### Physical Activity Variables

The variables related to time spent on physical activity and activity habits were categorized according to the scheme shown in Table 2.

In accordance with the World Health Organization (WHO) recommendations on physical activity [22], respondents engaging in less than 75 min of physical activity per week were classified as having ‘insufficient high-intensity activity’ while those engaging in more than 75 min per week were classified as having ‘healthy high-intensity activity’. For moderate-intensity activity, respondents performing less than 150 min per week were classified as having ‘insufficient moderate-intensity activity’, whereas those engaging in more than 150 min per week were categorized as having ‘healthy moderate-intensity activity’.

Sample socio-demographic collected included sex (analyzed in binary form, male and female), age (categorized as Young: 18–30 years, Adults: 31–65 years, and Seniors: >65 years), educational level (Basic education: no studies, only primary or secondary studies, vocational training or baccalaureate, and Higher education: degree, Master and PhD), Income level (Low income: <2200 €/month, Medium-high income: >2200 €/month and Not answer), municipality (<2000 inhabitants, 2000–10,000 inhabitants, and >10,000 inhabitants), living situation (living alone, living with others) and family living (living in the family home, living outside).

To assess potential indicators of eating disorders, this study included variables measuring worry about gaining weight or feeling overweight, difficulty controlling food intake or experiencing shame after eating, and body image concerns. These frequency-based variables were assessed using a Likert scale ranging from 1 to 6, where 6 indicated “always”, 5 “very frequently”, 4 “frequently”, 3 “occasionally”, 2 “rarely”, and 1 “never”. Additionally, participants were asked if they had ever been diagnosed with an eating disorder.

### 2.6. Data Analysis

Data collected were entered into a Microsoft Excel database and carefully reviewed to correct errors and inconsistencies, with special attention to entry mistakes and outliers. Some variables were categorized or calculated based on others. To ensure data reliability, extreme BMI values (below 14 and above 40) were excluded. Once the dataset was cleaned and organized, it was transferred to Jamovi (Version 2.3.28.0) [23] for further statistical analysis. The normality of the sample was assessed using the Shapiro–Wilk test, which confirmed that none of the variables met normality assumptions. Q-Q plots further validated this finding [24]. Due to the non-normal distribution of data, categorical variables were analyzed using the Chi-Square Test. Ordinal or numerical independent variables were analyzed using the Mann–Whitney U test for comparisons between two groups or the Kruskal–Wallis test for comparisons between three or more groups. The significance level was set at 0.05. Discrete variables are presented as absolute values and percentages, while continuous variables are reported as mean and standard deviation.

As an unsupervised machine learning approach, Principal Component Analysis (PCA) was applied to reduce dataset dimensionality. This method consolidates a large set of variables into fewer principal components that capture most of the original variance. A PCA plot allows for visualizing patterns in data, identifying clusters of observations, and understanding the influence of the original variables on the principal components. In a PCA graphical diagram, the arrows represent the original variables and their influence on the principal components. Longer arrows indicate that the variable contributes more to the variance explained by that axis, while shorter arrows suggest a lower influence on that component. The direction of the arrow indicates how the variable affects the principal component. Variables whose arrows point in a similar direction are positively correlated, whereas those with opposite directions are negatively correlated. If an arrow is perpendicular to an axis, it means that the variable does not contribute significantly to that principal component. Similarly, if an arrow is perpendicular to another arrow, it indicates that those variables are not related to each other.

## 3. Results

### 3.1. Socio-Demographic Characteristics

Approximately two-thirds of the participants were women, with a mean age of 39.9 years. Women were slightly older on average than men (40.6 vs. 38.6 years). Young individuals (18–30 years) accounted for 36.4% of the sample, adults (31–65 years) made up 61.3%, and only 2.3% were aged over 65. Most participants (58.1%) had higher education qualifications, lived with others (88.9%), and a significant proportion (78.9%) resided with family members. The detailed socio-demographic characteristics of the sample are outlined in Table 3.

### 3.2. Health Variables and Nutritional and Lifestyle Habits

The second column of Table 6 presents the mean scores with the standard deviation for various health variables, nutritional habits, and lifestyle behaviors. The average BMI of the sample was 24.2 ± 4.26, classifying the population within the normal weight range. Participants generally rated their self-perceived health positively, with a mean score of 3.98 out of 5. Regarding potential indicators of eating disorders, responses clustered around a score of 3, suggesting that concerns about weight gain, lack of control over food intake, or dissatisfaction with body image were only occasionally experienced.

Alcohol and tobacco consumption was relatively low, with most respondents consuming alcohol less than once per week and either not smoking or only smoking sporadically. Sleep duration averaged between 6 and 7.5 h per night, and participants frequently reported waking up feeling rested, with sleep quality ratings between 3 and 4 out of 5.

Examining food and drink consumption frequency, fruit intake (2.35 ± 0.75) ranged between one and four pieces per day, while vegetable consumption (3.45 ± 0.75) varied from a minimum of two portions per week to a maximum of one portion per day. Consumption of both white fish (1.72 ± 0.57) and blue fish (1.84 ± 0.59) was relatively low, with intake ranging from never to twice a week. White meat consumption (2.54 ± 0.68) was more frequent than red meat (1.74 ± 0.69). Unhealthy food consumption, including fast food (2.43 ± 0.76), fried food (2.32 ± 0.78), and ultra-processed food (2.31 ± 0.91), was relatively infrequent, occurring approximately two to four times per month. Regarding beverages, the consumption of sugary drinks (1.43 ± 0.66), juices (1.23 ± 0.54), and energy drinks (1.06 ± 0.30) was minimal, typically limited to a few times per month. Coffee consumption was more frequent, averaging around two cups per day (1.76 ± 0.71).

### 3.3. Physical Activity Habits

Table 4 presents the mean and standard deviation of respondents’ physical activity habits. On average, participants engage in high-intensity (1.05 ± 1.55) and moderate-intensity (1.33 ± 1.72) physical activity at work approximately one day per week, with an average duration of about 45 min per session. In contrast, active commuting is more common, as respondents tend to walk or cycle to work nearly every weekday (4.15 ± 2.55), spending an average of 44.9 ± 83.9 min on their journey.

During leisure time, participants engage in high-intensity physical activity an average of 2.41 ± 2.08 days per week and moderate-intensity activity 2.63 ± 2.06 days per week. The average duration of high-intensity exercise is 50.8 ± 55.9 min, while moderate-intensity exercise lasts longer, averaging 68.9 ± 276 min.

When combining physical activity from work, commuting, and leisure time, the total weekly duration amounts to 96.7 ± 208 min of high-intensity activity and 118 ± 337 min of moderate-intensity activity.

### 3.4. Physical Activity Habits in Relation to Socio-Demographic Variables

Table 5 and Table A3 in Appendix B present a comparison of physical activity habits across different socio-demographic groups. These data reveal significant differences in physical activity levels based on gender, age, education, and income. Men engaged in more intense physical activity at work (61.4 vs. 38.3 min/day) and spent more time in high-intensity sports (129 vs. 81 min/week) compared with women, who exhibited higher levels of sedentary behaviors (313 vs. 203 min/day). Age-related differences were also notable, with younger individuals being significantly more active in high-intensity sports (58.4 min/week) than seniors (18.6 min/week). Additionally, moderate-intensity activity was much lower in seniors (53.4 min/week) compared with adults (130 min/week).

Education and income also influenced activity patterns. Individuals with lower education levels engaged in more intense work-related physical activity (63.3 vs. 33.3 min/day), yet this was not compensated by increased leisure time exercise. Similarly, those with lower-income individuals engaged in more intense work-related activity (58.3 vs. 39.8 min/day) but did not participate in sports at higher rates. Social factors also played a role in physical activity habits. Participants living with family members walked or cycled more frequently (57.9 vs. 41.5 min/week) and engaged in more high-intensity sports (58.0 vs. 48.9 min/week) compared with those living alone. Overall, the findings suggest that sedentary behavior is more prevalent among women, seniors, and individuals with higher education or income, emphasizing the need for targeted interventions to promote physical activity within these groups. Regarding dietary patterns, we have included relevant analyses comparing participants following the Mediterranean diet and other dietary types, which can now be found in Appendix B.

### 3.5. Health and Lifestyle Variables and Their Relationship to Physical Activity

The statistical analysis reveals distinct differences between groups based on physical activity levels and lifestyle habits (Table 6), supported by significant mean values and *p*-values. Individuals engaging in healthy high-intensity activity (HHIA) reported better self-perceived health (mean 4.10 ± 0.72) compared with those with insufficient high-intensity activity (IHIA, mean 3.88 ± 0.77, *p* < 0.001). This finding underscores the positive association between regular high-intensity physical activity and perceived health status.

Psychological aspects also differed. HHIA participants had lower obesophobia scores (mean 2.86 ± 1.30 vs. 3.14 ± 1.35, *p* < 0.001) and greater self-control (mean 2.32 ± 1.08 vs. 2.49 ± 1.17, *p* = 0.010) than their IHIA counterparts. Body image was notably better among HHIA participants (mean 3.03 ± 1.24 vs. 3.22 ± 1.28, *p* = 0.003), a pattern also observed among individuals with healthy moderate-intensity activity (HMIA, mean 2.92 ± 1.24 vs. IMIA, mean 3.18 ± 1.26, *p* = 0.004).

Alcohol-related behaviors showed mixed results. While alcohol consumption did not differ significantly, HHIA participants were more likely to report episodes of getting drunk (mean 1.20 ± 0.53 vs. IHIA mean 1.13 ± 0.44, *p* = 0.003). Smoking was more common in IMIA individuals (mean 1.44 ± 0.87 vs. HMIA mean 1.29 ± 0.69, *p* = 0.006), while HHIA participants reported slightly longer sleep durations (*p* = 0.032).

Dietary patterns further highlighted distinctions. HHIA participants consumed fewer ultra-processed foods (mean 2.24 ± 0.89 vs. IHIA mean 2.36 ± 0.92, *p* = 0.008) and had lower dairy intake (mean 3.30 ± 0.95 vs. IHIA mean 3.39 ± 0.93, *p* = 0.026). However, HHIA individuals consumed more energy drinks (mean 1.10 ± 0.39 vs. IHIA mean 1.03 ± 0.20, *p* < 0.001) and coffee (mean 1.81 ± 0.72 vs. IHIA mean 1.72 ± 0.69, *p* = 0.013).

### 3.6. Principal Component Analysis

Given the large number of variables that influence health, nutrition, and lifestyle habits, a Principal Component Analysis (PCA) was utilized to reduce the dimensionality of the model.

**Figure 1:**
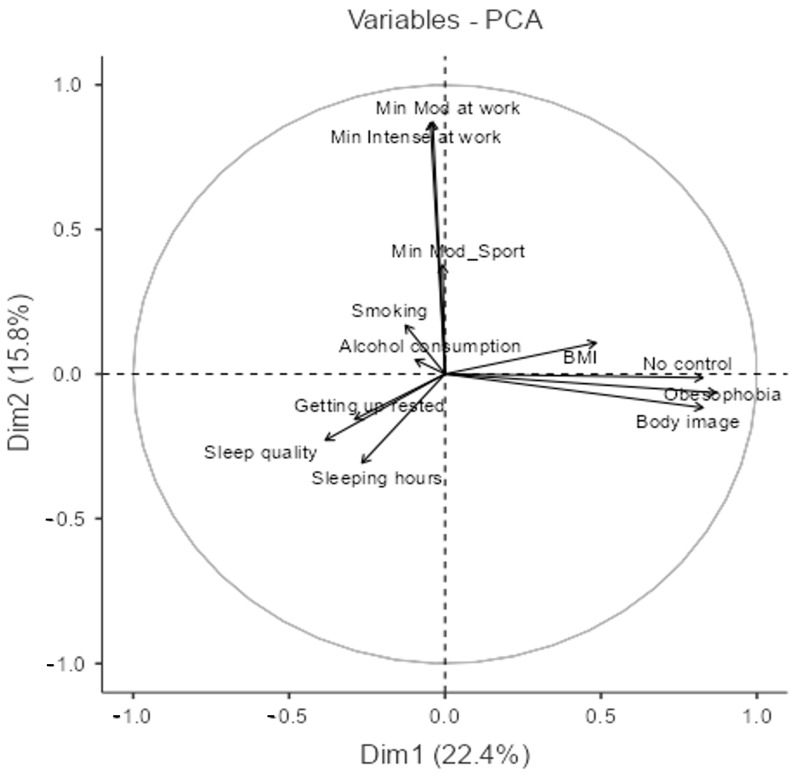
Variables plot of the PCA model between health and lifestyle habits variables and physical activity. (NOTE: Only the top twelve variables contributing the most to the PCA model are represented).

First, we tried to observe the relationship between health and lifestyle habits variables and physical activity (Figure 1); we chose two components, which explain 38.2% of the variance of these data (the percentage of variance explained by each component is reflected in Table A1 of Appendix A).

The PCA biplot (Figure 1) shows the relationships between twelve variables, with Dim1 (22.43%) and Dim2 (15.75%). Together, these two dimensions capture a meaningful portion of the dataset’s variability, allowing us to identify key patterns and associations.

Variables such as “Obesophobia”, “Body image”, “No control”, and “BMI” are strong. This Principal Component Analysis (PCA) biplot visually represents the relationships between various behavioral and psychological variables, with Dim1 explaining 22.4% of the variance and Dim2 accounting for 15.8%.

The length of the arrows indicates the strength of each variable’s contribution to the principal components. Longer arrows suggest that the variable is well-represented in the current dimensions, while shorter arrows indicate weaker associations or that the variable may be more relevant in other dimensions not displayed here. For instance, “Min Mod at work” and “Min Intense at work” have relatively long arrows extending along Dim2, highlighting their strong contribution to this component. Similarly, “Body image”, “Obesophobia”, and “No control” extend along Dim1, showing their importance in differentiating individuals based on body perception factors.

The angles between the arrows provide insights into the relationships between variables. Acute angles indicate a positive correlation, meaning the variables tend to increase or decrease together. For example, “Body image” and “Obesophobia” are closely aligned, suggesting a strong association between these factors. Similarly, “Sleep quality”, “Sleeping hours”, and “Getting up rested” form a cluster with relatively small angles, reinforcing their interdependence. In contrast, obtuse or nearly perpendicular angles suggest little to no correlation or even a potential inverse relationship. For instance, sleep-related variables are nearly orthogonal to body perception variables, implying that better sleep quality and longer sleep duration are not directly related to concerns about body image and weight.

Interestingly, smoking and alcohol consumption have relatively short arrows, suggesting that these behaviors may not be as strongly represented in the first two dimensions or that they contribute to variance in a more complex way. Their angles with other variables are relatively small with respect to each other, indicating a possible relationship between these two habits, but they do not show strong alignment with the main axes, meaning they may not be primary differentiators in this analysis.

Overall, this PCA biplot reveals distinct behavioral and psychological dimensions, where physical activity strongly aligns with Dim2, while body perception factors dominate Dim1. Sleep quality and duration, positioned in a different quadrant, appear to represent an independent aspect of lifestyle, suggesting a complex interplay between health behaviors and psychological well-being.

**Figure 2:**
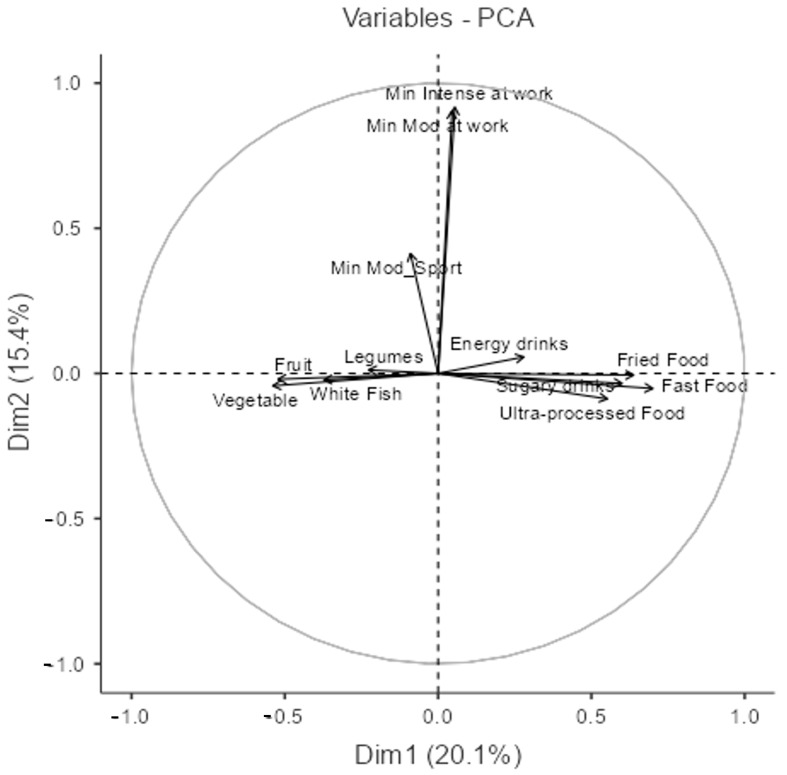
Variables plot of the PCA model between the frequency of consumption of certain food groups and physical activity. (NOTE: Only the top twelve variables contributing the most to the PCA model are represented).

Second, we explored the relationship between the frequency of consumption of certain food groups and the time devoted to physical activity (Figure 2); we chose two components, which explain 35.5% of the variance of these data (the percentage of variance explained by each component is reflected in Table A2 of Appendix A).

The PCA biplot (Figure 2) displays the relationships between variables associated with dietary habits and physical activity, with Dim1 (20.08%) and Dim2 (15.37%).

This PCA biplot reveals distinct patterns in dietary habits and physical activity behaviors. The first dimension (Dim1) appears to contrast healthy and unhealthy eating habits. On one side, foods such as “Fruit”, “Vegetable”, “Legumes”, and “White Fish” cluster together, suggesting that individuals who consume one of these tend to consume the others as well. On the opposite side, “Ultra-processed Food”, “Fast Food”, and “Fried Food” form a strong grouping, indicating that these unhealthy food choices are also frequently consumed together. Additionally, “Sugar-sweetened beverages” and “Energy drinks” are positioned near unhealthy foods, implying that those who consume processed foods and fast food are also more likely to drink sugary beverages.

In contrast, the second dimension (Dim2) is largely associated with physical activity. “Min Intense at work” and “Min Mod at work” are strongly aligned in the same direction, suggesting that individuals who engage in one form of work-related physical activity also tend to engage in the other. Similarly, “Min Mod Sport” is somewhat separate from work-related activity but still indicates a relationship between movement and exercise.

Notably, dietary habits and physical activity behaviors seem to be largely independent of one another, as suggested by the separation of food-related variables along Dim1 and physical activity variables along Dim2. This means that individuals with higher levels of work-related physical activity do not necessarily have healthier or unhealthier eating patterns, reinforcing the idea that diet and physical activity are distinct lifestyle factors. However, the fact that “Energy drinks” are positioned relatively close to physical activity variables suggests a potential association, possibly indicating that individuals who engage in more intense work activity tend to consume energy drinks more frequently.

Overall, this PCA suggests that eating habits form two well-defined groups—healthy and unhealthy—while physical activity at work and sports participation exist as separate lifestyle dimensions with little direct association with dietary choices.

## 4. Discussion

This study analyzes a sample of the Spanish adult population, with particular attention to women and young individuals, revealing significant socio-demographic patterns and health behaviors. Key findings highlight the influence of factors such as sex, age, education, and family structure on physical activity levels, as well as the complex relationship between physical activity, dietary habits, and psychological well-being. The results underscore the need for integrated health promotion strategies that address both physical activity and nutrition to enhance overall well-being.

The sample shows a female prevalence of 67% and an average age of 39.9 years, with only 2.3% of participants over 65, which limits the analysis of older populations. Additionally, 58.1% of respondents have a higher education level, and 52% report middle-high income, while 36.6% indicate low income, reflecting economic disparities that may influence participation. The average BMI is 24.2, indicating good general health, and the self-perceived health score averages 3.98 out of 5. Eating disorders appear to be uncommon, and alcohol and tobacco use is low. Participants report sleeping between 6 and 7.5 h per night, with moderate sleep quality. Dietary patterns reveal moderate consumption of fruits and vegetables but insufficient fish intake. Physical activity is limited, with an average of 1.05 days of intense activity at work and 4.15 days of active mobility. Although the sample meets the recommended levels for moderate physical activity, vigorous activity remains underutilized [22]. Overall, the participants demonstrate good health behaviors, although there is room for improvement, especially regarding physical activity and diet.

The analysis of physical activity patterns according to socio-demographic variables reveals noteworthy trends. Physical activity is influenced by sex, age, education level, income, and family structure.

Comparisons between men and women indicate that men tend to be more active, particularly in high-intensity work activities and vigorous sports. Specifically, men report an average of 61.4 min per day of intense physical activity at work compared with 38.3 min for women, and they engage in high-intensity sports for 129 min per week versus 81 min for women. Conversely, women tend to report more sedentary behavior, with an average of 313 min of rest per day compared with 203 min for men. These findings are consistent with previous studies indicating that men generally engage in more intense physical activities, particularly in occupational contexts [25]. The literature also suggests that while women participate less in vigorous physical activity, they often compensate through moderate-intensity activities such as walking or cycling [26].

To address these gender disparities, it is crucial to develop physical activity programs specifically designed for women, emphasizing accessible and motivating high-intensity options. Additionally, modifying work environments to reduce sedentary time—such as implementing standing desks and promoting active breaks—can increase overall activity [27]. Experimental studies have shown that such measures can improve physical activity levels and reduce health risks associated with sedentary behavior, including cardiovascular diseases and type 2 diabetes [27]. Furthermore, awareness campaigns should target both genders, promoting the integration of high-intensity activity into everyday life [27].

Significant differences also emerge across age groups. Young adults are more active in high-intensity sports, with an average of 58.4 min per week compared with just 18.6 min among older adults. Their weekly moderate activity levels are also higher (53.3 vs. 22.4 min). These findings align with previous research showing that physical activity tends to decrease with age because of factors such as reduced mobility and changes in lifestyle and social roles [28]. Motivational aspects are also key, as young people are often more engaged in active lifestyles, while older individuals may prefer less demanding activities or avoid exercise because of health constraints [28].

To support physical activity at all life stages, it is essential to design age-appropriate programs, including low-impact options for older adults to enhance mobility and health. Studies such as Paterson et al. (2007) have shown that activities such as tai chi, swimming, and walking significantly improve balance, strength, and cardiovascular health in older populations [29]. At the same time, maintaining engagement among younger individuals can be encouraged through strategies such as discounted gym memberships and the promotion of safe, accessible public spaces for intergenerational activity [29].

Educational and income-related disparities are also notable. Participants with lower education and income levels engage more frequently in physical activity at work (63.3 min vs. 33.3 min for those with higher education) but are less likely to engage in physical activity during their leisure time. This suggests that while these individuals may perform more physically demanding jobs, they face barriers to additional recreational activity, likely because of financial limitations or time constraints. The existing literature supports these findings, indicating that individuals with lower education and income are less likely to engage in leisure-time physical activity, primarily because of a lack of access to suitable facilities and limited availability of free time [30].

To counteract these inequalities, it is essential to ensure free or low-cost access to sports facilities for lower-income populations. Additionally, implementing physical activity programs within workplaces can help balance occupational demands with healthy movement. Targeted educational campaigns are also needed to raise awareness about the importance of engaging in physical activity during leisure time.

Another relevant factor is family life. These data show that individuals living with family members are more physically active in activities such as walking or cycling (57.9 min compared with 41.5 min for those living alone), as well as in high-intensity sports (58 min compared with 48.9 min). This can be attributed to the fact that cohabitation—especially with children or relatives—often encourages participation in daily physical activities. Some studies suggest that families, particularly those with children, can promote physical activity through shared routines and commitments [31]. Moreover, having a partner or children can serve as motivation to prioritize health and physical well-being.

To reinforce this positive trend, it is beneficial to develop initiatives and sports programs that involve the entire family, encouraging joint activities between parents and children. Children of physically active parents are more likely to adopt similar habits, emphasizing the importance of family role models [31]. Research indicates that family-based sports programs improve adherence to physical activity in both children and adults. For instance, the “Families on the Move” program significantly increased physical activity while reducing sedentary behavior [32]. Shared activities—such as walking, outdoor games, or team sports—not only boost physical activity levels but also enhance family bonds and psychological well-being [33].

In addition, urban policies that support the creation of safe, accessible public spaces for outdoor physical activity are crucial for fostering active lifestyles. Studies show that the presence of parks, cycling lanes, and pedestrian areas significantly increases physical activity in the general population, helping to prevent chronic conditions associated with sedentary behavior [30]. Neighborhoods equipped with active mobility infrastructures (e.g., bike paths, wide sidewalks, and safe play areas) report higher rates of physical activity, particularly among families with children [30]. A successful example is the “Safe Routes to School” project, which promoted walking and cycling among schoolchildren while simultaneously improving safety and air quality in school zones [30].

Finally, offering incentives such as discounted family gym memberships or group sports classes can transform physical activity into a social and community-oriented experience, amplifying the health benefits for all family members. Financial incentive policies have shown positive outcomes in increasing physical activity participation. For example, family discounts on gym memberships have been linked to more than a 20% increase in participation in certain urban areas [34]. Furthermore, programs that combine exercise with social interaction—such as group classes for parents and children—promote sustained engagement and reduce dropout rates over time [34].

The analysis of these data reveals a clear association between levels of physical activity and self-perceived health. Participants engaging in healthy high-intensity physical activity (HHIA) report a significantly better perception of their health (mean = 4.10 ± 0.72) compared with those with insufficient high-intensity activity (IHIA, mean = 3.88 ± 0.77), with a statistically significant difference (*p* < 0.001). This finding aligns with numerous studies indicating that higher physical activity levels are linked to more positive health perceptions [6,35,36]. The literature widely documents the positive effects of physical activity on quality of life and psychological well-being [6,37,38]. Regular physical activity—particularly at high intensity—has been associated with benefits for both physical and mental health. Active individuals tend to perceive their health more favorably than sedentary ones, likely because of greater energy levels, improved physical functioning, and an enhanced sense of well-being [36].

Beyond self-perception of health, physical activity plays a critical role in reducing symptoms of depression and anxiety. Several studies have shown that exercise, particularly aerobic activity, stimulates endorphin release and helps regulate stress, thereby improving mood and psychological resilience [37,38]. Another important benefit is improved sleep quality. Regular physical activity has been linked to deeper, more restorative sleep, reduced insomnia symptoms, and improved sleep efficiency [39], which is essential for both physical and mental health. Additionally, physical activity has demonstrated positive effects on cognitive function. Aerobic exercise, in particular, has been associated with improvements in memory, attention, and processing speed, as well as reduced risk of cognitive decline and dementia in older adults [39]. These findings confirm that physical activity contributes not only to physical health but also to psychological and cognitive well-being, ultimately enhancing overall quality of life.

Regarding obesophobia, body image, and self-control, these data suggest that individuals engaging in HHIA exhibit significantly lower levels of obesophobia and a more positive body image compared with other groups. Furthermore, HHIA participants demonstrate greater self-control. These results are consistent with existing research on the psychological benefits of physical activity, including improved self-regulation and body image [40,41]. Regular exercise appears to promote greater body awareness and better emotional and behavioral regulation [42]. Additionally, it fosters self-efficacy and enhances individuals’ sense of control over their health, which may mitigate feelings of bodily insecurity often exacerbated by unhealthy lifestyle habits [42].

Substance use behaviors follow more complex patterns. While no significant differences in overall alcohol consumption were found between groups, individuals engaging in intense physical activity tend to consume alcohol more occasionally. In contrast, smoking was significantly more prevalent among those with insufficient moderate physical activity (IMIA), reinforcing the inverse relationship between physical activity and tobacco use [43].

Notably, participants in the HHIA group report longer sleep durations than individuals in other groups. Multiple studies support the idea that physical activity—particularly at higher intensities—enhances sleep quality and recovery, lowering the risk of sleep disorders [44,45,46]. Exercise has been shown to improve sleep efficiency and reduce sleep onset latency, thereby contributing to better overall rest [44].

Analysis of dietary patterns reveals significant differences based on physical activity levels. In general, individuals in the HHIA group tend to follow more balanced diets than those in the IHIA group. One of the most notable findings is the reduced consumption of ultra-processed foods in the HHIA group (2.24 ± 0.89 vs. 2.36 ± 0.92; *p* = 0.008), suggesting a stronger preference for natural food sources. This pattern is consistent with studies showing that active individuals are more likely to follow diets rich in unprocessed foods [47].

Dairy product consumption was also slightly lower among more physically active individuals (3.30 ± 0.95 vs. 3.39 ± 0.93; *p* = 0.026), possibly because of preferences for alternative protein sources such as plant-based options or protein supplements, which are often used to support performance and recovery [48]. Conversely, individuals in the HHIA group consumed more energy drinks (1.10 ± 0.39 vs. 1.03 ± 0.20; *p* < 0.001) and coffee (1.81 ± 0.72 vs. 1.72 ± 0.69; *p* = 0.013), indicating a potential reliance on stimulants to sustain elevated energy demands. These findings are supported by the literature highlighting increased caffeine consumption among athletes for improved endurance and concentration [49].

Similarly, individuals with healthy moderate-intensity physical activity (HMIA) showed some dietary differences compared with those with insufficient moderate-intensity activity (IMIA), though to a lesser extent. Legume consumption was slightly higher among the HMIA group (2.07 ± 0.63 vs. 1.98 ± 0.58; *p* = 0.045), suggesting a higher intake of plant-based proteins. Epidemiological studies have linked this type of protein to better body composition and a lower prevalence of metabolic disorders [50]. Fast food consumption was marginally lower in more active individuals (2.36 ± 0.71 vs. 2.45 ± 0.77; *p* = 0.050), consistent with findings that active lifestyles correlate with healthier eating habits [51].

No significant differences were found across groups regarding the consumption of cereals, fruits, vegetables, meats, fish, or sugary drinks, suggesting that some dietary components may be less influenced by activity level. Overall, these data suggest that physically active individuals—especially those engaging in high-intensity activity—are more likely to follow healthier diets with lower consumption of ultra-processed and fast foods. However, they also tend to consume more energy drinks and coffee, likely in response to the physiological demands of intense exercise. These findings highlight the need for a balanced approach that promotes both physical activity and healthy nutrition.

To further explore the interplay between health variables, nutrition, and lifestyle, two Principal Component Analyses (PCAs) were conducted. This statistical method reduces complexity by identifying underlying patterns and associations. The first PCA examined the relationships among health, lifestyle, and physical activity variables, while the second focused on the frequency of food group consumption and time spent on physical activity.

The first PCA identified two principal components explaining 38.2% of the variance. The biplot illustrates the distribution of twelve key variables, with Dimension 1 (Dim1) explaining 22.43% of the variance and Dimension 2 (Dim2) explaining 15.75%. Dim1 is mainly associated with psychological variables related to body image, including “Obesophobia”, “Body Image”, and “No Control”. These findings suggest that individuals with negative body image perceptions often engage in specific behaviors aimed at weight control [52], consistent with previous research linking body dissatisfaction to disordered eating [53]. Dim2 is primarily related to physical activity, with variables such as “Minutes of Moderate Activity at Work” and “Minutes of Intense Activity at Work” contributing significantly. This supports findings that individuals engaging in one form of occupational activity tend to participate in others as well. The inclusion of workplace physical activity in overall activity levels is supported by evidence of its benefits for cardiovascular and metabolic health [6].

Interestingly, sleep duration and quality appear to be relatively independent of the other assessed variables, positioned in a distinct quadrant. Alcohol and tobacco use are represented by short vectors in the biplot, indicating that these behaviors contribute minimally to the explained variance. This supports the existing literature highlighting the fundamental role of sleep in physical and mental health and its inverse association with obesity and metabolic disorders [54].

The second PCA explored the relationship between food group consumption and physical activity time. Dim1 (20.08% of variance) separates healthy from unhealthy dietary patterns. Foods such as “Fruit”, “Vegetables”, “Legumes”, and “White Fish” cluster together, reflecting a healthier dietary profile, in line with studies linking such diets to reduced chronic disease risk [36]. In contrast, “Ultra-Processed Food”, “Fast Food”, and “Fried Food” form a distinct cluster indicative of poorer dietary habits, which are known risk factors for obesity and cardiovascular diseases [47].

Dim2, primarily associated with physical activity, includes variables such as “Minutes of Intense Activity at Work” and “Minutes of Moderate Activity at Work”, confirming that engagement in one type of activity often correlates with another. However, the limited overlap between food and activity variables suggests that dietary habits and physical activity operate largely independently. This is consistent with findings indicating that, while both are crucial to health, they may be influenced by distinct behavioral determinants [52]. Notably, “Energy Drinks” is positioned close to physical activity variables in the biplot, suggesting a potential association between higher physical effort and increased consumption of such beverages. This may reflect physiological demands, such as electrolyte and fluid replacement, following intense exercise [55].

In addition, cultural and social influences—particularly those promoted on social media—have contributed to the popularity of energy drinks among active individuals. Despite their positioning as performance enhancers, these beverages often contain high levels of sugar, caffeine, and other stimulants, which may carry health risks if consumed excessively [56]. Thus, while energy drinks may offer perceived short-term benefits, their widespread and sometimes uncritical use raises important concerns from a public health standpoint.

Overall, the PCA analyses offer a comprehensive perspective on the interrelations among health, lifestyle, and nutrition variables. The separation of components related to physical activity, diet, and body perception underscores the need for targeted health strategies that address these factors independently. These findings reinforce the idea that although physical activity and nutrition are both essential for health, they may follow distinct behavioral pathways influenced by different underlying factors.

### 4.1. Strengths and Limitations

One of the key strengths of this study lies in its large sample size and the geographic breadth of data collection, which spans the entire Spanish territory. This allows for a comprehensive analysis of the socio-demographic, nutritional, and lifestyle factors influencing physical activity. The use of the validated Nutritional and Social Scale of Healthy Habits (NutSo-HH) ensures high-quality and reliable data collection. Moreover, the integration of traditional statistical techniques with advanced analytical methods, such as Principal Component Analysis (PCA), enhances the robustness of the findings. The application of PCA to explore psychological and behavioral dimensions offers deeper insights into the complex relationships between diet, physical activity, and overall health, providing a more nuanced understanding of well-being dynamics.

Despite these strengths, this study also presents several limitations that must be acknowledged. First, the cross-sectional design precludes the ability to infer causal relationships between lifestyle factors and physical activity. Future research would benefit from a longitudinal approach to assess the evolution of these patterns over time.

Another limitation is the reliance on self-reported data, which may be subject to response and recall biases, potentially affecting the accuracy of the results. The sampling method, which primarily employed snowball sampling and dissemination through social media, also introduces self-selection bias. This approach, while effective in increasing participation, allows initial respondents to influence the sample’s composition. The involvement of social media influencers in disseminating the survey may have further shaped respondent profiles by attracting individuals with similar interests and behaviors.

A notable limitation of this study lies in the sampling imbalance. The sample was predominantly composed of women (67%), younger individuals (36.4% aged 18–30), and participants with higher educational attainment (58.1%). These characteristics reduce the generalizability of the findings and may reflect a participation bias commonly observed in health-related research, where women and younger, more educated individuals tend to be overrepresented [57,58,59]. Furthermore, the use of non-probabilistic snowball sampling through social media may have excluded older adults, less tech-savvy individuals, and those from lower-income groups, potentially skewing the results. In particular, only 2.3% of participants were aged over 65, which significantly limits this study’s ability to explore age-related health behaviors—especially those critical to chronic disease prevention and healthy aging.

To mitigate these biases, several strategies were employed to broaden the sample’s diversity. The questionnaire was distributed in physical locations across Spain, with posters featuring QR codes placed in community centers, markets, and local businesses to reach populations with limited online access. Additionally, email outreach targeted a variety of organizations, including homemaker associations, scout groups, and charities, to encourage broader demographic participation. A new round of data collection is currently underway with the aim of improving balance in terms of age, gender, and socioeconomic representation, thereby enhancing the robustness and representativeness of future findings.

Finally, another limitation of this study is the reliance on Body Mass Index (BMI) as an indicator of nutritional status. While BMI is a widely used and accessible measure in epidemiological studies, it lacks sensitivity in distinguishing between different components of body composition—such as muscle mass and fat mass—which may lead to the misclassification of metabolically healthy individuals as overweight or obese. This is particularly relevant in populations with higher levels of physical activity or varying body types, where BMI may not accurately reflect cardiometabolic risk. Future studies would benefit from including more precise measures of body composition, such as waist circumference, body fat percentage, or bioelectrical impedance analysis, to improve the assessment of nutritional and health status.

### 4.2. Areas for Further Research

Future research should aim to include a larger number of elderly participants to gain a more comprehensive understanding of their health and well-being dynamics while also addressing the specific barriers that may limit their participation. Additionally, further studies could explore the impact of public policies and awareness campaigns on health behaviors—particularly in relation to alcohol and tobacco consumption—offering insights into the effectiveness of these interventions.

Given the observed complexity of the relationship between physical activity and dietary choices, it is essential to investigate how psychological and social factors jointly influence both behaviors. In this context, future research could also assess the effectiveness of targeted interventions designed to promote active mobility in urban settings as a means to enhance public health and combat sedentary lifestyles.

Another important area of exploration involves understanding how cultural and social factors shape health behaviors and physical activity, especially across different age groups and socioeconomic strata. Moreover, the relationship between physical activity and mental health warrants deeper investigation, with particular emphasis on how physical activity can serve as an intervention to improve psychological well-being across diverse populations.

Finally, longitudinal studies are crucial for monitoring changes in health behaviors over time and identifying the factors that influence the long-term sustainability of healthy lifestyles. These lines of inquiry will contribute to a more integrated and comprehensive understanding of population health, offering valuable guidance for the development of future public health strategies and policies.

## 5. Conclusions

This analysis underscores the intricate interplay between health behaviors, diet, and physical activity, revealing that while these factors are interrelated, they often follow distinct trajectories. The sample examined demonstrates generally positive lifestyle habits, including a normal body mass index, favorable self-perceived health, and moderate levels of alcohol and tobacco use. Nonetheless, certain areas—particularly dietary habits—present opportunities for improvement, such as increasing the consumption of fish and reducing red meat intake.

Although physical activity levels are adequate overall, the intensity of exercise tends to fall short of official health recommendations. This points to the need for strategies that encourage more vigorous physical activity, both during leisure time and within the workplace. These data also reveal that physical activity is significantly influenced by socio-demographic variables such as sex, age, educational attainment, income, and family structure. These findings highlight the importance of tailoring public health policies to address these disparities and ensure equitable access to healthy lifestyle opportunities.

A key takeaway from this study is the complexity of the relationship between physical activity and dietary behaviors. While both are fundamental to health, they appear to be shaped by distinct determinants, suggesting that integrated interventions—targeting both areas simultaneously—may be more effective than those addressing them in isolation. Moreover, the independent positioning of sleep-related variables in the PCA biplot emphasizes the importance of considering sleep quality and duration as critical components of holistic well-being strategies.

Overall, the findings reinforce the necessity of adopting multidimensional approaches in public health promotion. To further improve intervention effectiveness and deepen our understanding of behavioral dynamics, future research should incorporate longitudinal data to establish causal relationships among lifestyle factors. Integrating these insights into policy-making and health education programs could play a pivotal role in promoting healthier, more sustainable behaviors across the population.

## Figures and Tables

**Table 1:** Categorization of the health and lifestyle variables.

Variable	Category	Score
Sleeping hours	<6 h	1
	6–7 h	2
	7–8 h	3
	>8 h	4
Getting up rested	Never	1
	Very seldom and sometimes	2
	Frequently and almost always	3
	Always	4
Sleep quality	0 and 1	1
	2	2
	3	3
	4 and 5	4
Water	Never, very rarely (2 max. per month), 1 glass/cup/week, and 2 or more glasses/cups/week	1
	2 glasses/cups or less every day	2
	3 to 5 glasses every day	3
	>5 glasses every day	4
Sugary soft drinks, coffee, and energy drinks	Never and very rarely (2 glasses max. per month)	4
	1 glass per week and ≥2 glasses per week	3
	≤2 glasses every day	2
	3 to 5 glasses and >5 glasses every day	1
Juice	Never and very rarely (2 glasses max. per month)	1
	1 glass per week and ≥2 glasses per week	2
	≤2 glasses every day	3
	3 to 5 glasses and >5 glasses every day	4
Fish consumption	Never or very seldom	1
	1–2 times a week	2
	≥3 times a week	3
	Every day	4
Consumption of fast food, fried, and ultra-processed dishes	Never	1
	Very seldom (2 times a month maximum)	2
	Once a week	3
	Several times a week	4
Getting drunk	Never or less than once a month	1
	Monthly	2
	Weekly	3
	Daily or almost daily	4
Alcohol consumption	Never or once a month	1
	2–4 times a month	2
	2–3 times a week	3
	4–5 times a week or every day	4
Smoking	Non-smoker	1
	Light smoker (<5 cigarettes per day)	2
	Moderate smoker (6–15 cigarettes per day)	3
	Severe smoker (>16 cigarettes per day)	4
Night outings	Never and sporadically	1
	1–2 times a week	2
	>3 times a week	3
	Every day	4
Sedentary lifestyle	<7 h	1
	7–9 h	2
	9–11 h	3
	>11 h	4

**Table 2:** Categorization of physical activity variables.

Variables	Categorization
At work or in the daily commute	
Days Intense physical activity at work	Likert scale 0–5
Minutes of Intense physical activity at work	Numerical
Days Moderate physical activity at work	Likert scale 0–5
Minutes Moderate physical activity at work	Numerical
Days a week Walking or cycling	Likert scale 0–7
Minutes a week Walking or cycling	Numerical
In leisure time	
Days a week High-intensity sport	Likert scale 0–7
Minutes a week High-intensity sport	Numerical
Days a week Moderate-intensity sport	Likert scale 0–7
Minutes a week Moderate-intensity sport	Numerical
Daily lying time (minutes)	Numerical
Total minutes at work or in the daily commute plus minutes in leisure time	
Total minutes a week High-intensity sport	Numerical
Total minutes a week Moderate-intensity sport	Numerical

**Table 3:** Sample and their socio-demographic characteristics (n = 1534).

	Mean (SD) or N (%)
Sex	
Male	506 (33.0%)
Female	1028 (67.0%)
Age (years)	39.9 (15.0)
Male Age (years)	38.6 (15.5)
Female Age (years)	40.6 (14.7)
Age (%)	
Young (18–30 years)	558 (36.4%)
Adults (31–65 years)	941 (61.3%)
Seniors (>65 years)	35 (2.3%)
Education level	
Basic education	642 (41.9%)
Higher education	892 (58.1%)
Income level	
Low	561 (36.6%)
Medium-high	797 (52.0%)
Don’t know-no answer	176 (11.5%)
Living situation	
Alone	171 (11.1%)
With others	1363 (88.9%)
Family living	
Without relatives	323 (21.1%)
With relatives	1211 (78.9%)

**Table 4:** Physical activity habits.

Variables	Mean	(SD)
At work or in the daily commute		
Days intense physical activity at work	1.05	(1.55)
Minutes of intense physical activity at work	45.9	(195)
Days moderate physical activity at work	1.33	(1.72)
Minutes of moderate physical activity at work	49.5	(139)
Days per week Walking or cycling	4.15	(2.55)
Minutes per week Walking or cycling	44.9	(83.9)
In leisure time		
Days per week of high-intensity sport	2.41	(2.08)
Minutes per week of high-intensity sport	50.8	(55.9)
Days per week of moderate-intensity sport	2.63	(2.06)
Minutes per week of moderate-intensity sport	68.9	(276)
Daily lying time (minutes)	276	(2820)
Total minutes at work or in the daily commute plus minutes in leisure time		
Total minutes a week High-intensity sport	96.7	(208)
Total minutes per week of moderate-intensity sport	118	(337)

**Table 5:** Comparison between physical activity habits and socio-demographic variables sex and age.

		Mean (SD)	Mean (SD)	Mean (SD)		
Sex	Sport Habits	Man	Women		*p*-Value *	
	At work or in the daily commute					
	Days Intense physical activity at work	1.46 (1.78)	0.85 (1.38)		<0.001 **	
	Minutes Intense physical activity at work	61.4 (210)	38.3 (187)		<0.001 **	
	Days Moderate physical activity at work	1.57 (1.77)	1.21 (1.68)		0.002 **	
	Minutes Moderate physical activity at work	55.8 (139)	46.4 (139)		0.223	
	Days a week Walking or cycling	4.04 (2.59)	4.21 (0.54)		0.122	
	Minutes a week Walking or cycling	48.3 (81.2)	43.3 (85.2)		0.473	
	In leisure time					
	Days a week High-intensity sport	3.11 (2.13)	2.07 (1.97)		<0.001 **	
	Minutes a week High-intensity sport	67.2 (63.7)	42.8 (49.7)		<0.001 **	
	Days a week Moderate-intensity sport	3.09 (2.06)	2.41 (2.02)		<0.001 **	
	Minutes a week Moderate-intensity sport	75.6 (272)	65.6 (293)		<0.001 **	
	Daily lying time (minutes)	203 (397)	313 (3434)		<0.001 **	
	Total minutes at work or in the daily commute plus minutes in leisure time					
	Total minutes a week High-intensity sport	129 (226)	81.0 (196)		<0.001 **	
	Total Minutes a week Moderate-intensity sport	131 (305)	112 (352)		<0.001 **	
Age		Y:Young	A:Adults	S:Senior	*p*-value ^$^	
	At work or in the daily commute					
	Days Intense physical activity at work	1.12 (1.60)	1.01 (1.51)	1.20 (1.73)	0.493	
	Minutes Intense physical activity at work	49.9 (203)	43.6 (194)	44.4 (72.9)	0.673	
	Days Moderate physical activity at work	1.42 (1.76)	1.27 (1.69)	1.49 (1.79)	0.258	
	Minutes Moderate physical activity at work	49.6 (141)	50.2 (140)	31.0 (48.2)	0.760	
	Days a week Walking or cycling	4.55 (2.36)	3.94 (2.63)	3.60 (2.76)	<0.001 **	Y vs. A: <0.001; Y vs. S: 0.106; A vs. S: 0.734
	Minutes a week Walking or cycling	46.9 (68.4)	43.8 (92.9)	42.9 (47.6)	0.022 **	Y vs. A: <0.016; Y vs. S: 0.948; A vs. S: 0.882
	In leisure time					
	Days a week High-intensity sport	2.82 (2.03)	2.21 (2.07)	1.31 (1.89)	<0.001 **	Y vs. A: <0.001; Y vs. S: <0.001; A vs. S: 0.018
	Minutes a week High-intensity sport	58.4 (52.4)	47.5 (58.0)	18.6 (23.8)	<0.001 **	Y vs. A: <0.001; Y vs. S: <0.001; A vs. S: 0.005
	Days a week Moderate-intensity sport	2.62 (2.03)	2.67 (2.07)	1.89 (2.07)	0.085	
	Minutes a week Moderate-intensity sport	53.3 (50.9)	79.9 (362)	22.4 (26.7)	<0.001 **	Y vs. A: 0.875; Y vs. S: <0.001; A vs. S < 0.001
	Daily lying time (minutes)	387 (4245)	213 (1510)	214 (280)	<0.001 **	Y vs. A: <0.016; Y vs. S: 0.996; A vs. S: 0.356
	Total minutes at work or in the daily commute plus minutes in leisure time					
	Total minutes a week High-intensity sport	108 (215)	213 (206)	214 (85.1)	<0.001 **	Y vs. A: <0.001; Y vs. S: 0.009; A vs. S: 0.161
	Total Minutes a week Moderate-intensity sport	103 (153)	130 (414)	53.4 (66.8)	0.002 **	Y vs. A: 0.998; Y vs. S: <0.001; A vs. S: 0.002

* Mann–Whitney-U test; ^$^ Kruskal Wallis test; ** Statistically significant.

**Table 6:** Health and lifestyle variables and their relationship to physical activity.

Healthy Variables and Nutritional and Lifestyle Habits	Total Population		Insufficient High-Intensity Activity (IHIA)	Healthy High-Intensity Activity (HHIA)		Insufficient Moderate-Intensity Activity (IMIA)	Healthy Moderate-Intensity Activity (HMIA)	
	Mean	(SD)	Mean (SD)	Mean (SD)	*p*-Value *	Mean (SD)	Mean (SD)	*p*-Value *
BMI	24.2	(4.26)	24.4 (4.69)	23.8 (3.65)	0.235	24.2 (4.32)	24.1 (4.01)	0.439
Self-perceived health	3.98	(0.75)	3.88 (0.77)	4.10 (0.72)	<0.001 **	3.96 (0.76)	4.04 (0.74)	0.08
Obesophobia	3.01	(1.34)	3.14 (1.35)	2.86 (1.30)	<0.001 **	3.04 (1.33)	2.87 (1.34)	0.064
No control	2.41	(1.14)	2.49 (1.17)	2.32 (1.08)	0.010 **	2.43 (1.14)	2.32 (1.13)	0.139
Body image	3.13	(1.26)	3.22 (1.28)	3.03 (1.24)	0.003 **	3.18 (1.26)	2.92 (1.24)	0.004 **
Getting drunk	1.16	(0.48)	1.13 (0.44)	1.20 (0.53)	0.003 **	1.16 (0.48)	1.16 (0.49)	0.866
Alcohol consumption	1.83	(0.87)	1.81 (0.87)	1.84 (0.88)	0.487	1.84 (0.86)	1.77 (0.92)	0.067
Smoking	1.32	(0.73)	1.32 (0.73)	1.32 (0.72)	0.547	1.29 (0.69)	1.44 (0.87)	0.006 **
Sleeping hours	2.41	(0.73)	2.41 (0.75)	2.41 (0.71)	0.821	2.43 (0.73)	2.32 (0.74)	0.032 **
Sleep quality	3.31	(0.85)	3.26 (0.90)	3.38 (0.77)	0.091	3.32 (0.85)	3.29 (0.83)	0.406
Getting up rested	2.6	(0.57)	2.59 (0.58)	2.61 (0.56)	0.479	2.60 (0.58)	2.62 (0.56)	0.599
Night outing	1.34	(0.52)	1.34 (0.53)	1.34 (0.51)	0.760	1.36 (0.53)	1.27 (0.45)	0.010 **
Fruit	2.35	(0.75)	2.32 (0.74)	2.38 (0.76)	0.171	2.35 (0.74)	2.34 (0.77)	0.693
Vegetable	3.45	(0.75)	3.46 (0.76)	3.44 (0.74)	0.265	3.45 (0.76)	3.45 (0.71)	0.614
White Fish	1.72	(0.57)	1.71 (0.57)	1.74 (0.55)	0.263	1.73 (0.57)	1.70 (0.53)	0.581
Blue Fish	1.84	(0.59)	1.82 (0.58)	1.86 (0.60)	0.202	1.84 (0.59)	1.83 (0.61)	0.777
White meat	2.54	(0.68)	2.51 (0.68)	2.57 (0.69)	0.121	2.55 (0.67)	2.50 (0.72)	0.128
Red meat	1.74	(0.69)	1.71 (0.67)	1.77 (0.72)	0.161	1.74 (0.69)	1.74 (0.71)	0.922
Dairy	3.35	(0.94)	3.39 (0.93)	3.30 (0.95)	0.026 **	3.36 (0.94)	3.32 (0.94)	0.426
Cereals	2.70	(1.01)	2.73 (1.00)	2.67 (1.01)	0.247	2.71 (1.00)	2.68 (1.03)	0.765
Legumes	2.00	(0.59)	1.99 (0.58)	2.02 (0.61)	0.233	1.98 (0.58)	2.07 (0.63)	0.045
Fast Food	2.43	(0.76)	2.44 (0.77)	2.43 (0.76)	0.840	2.45 (0.77)	2.36 (0.71)	0.050
Fried Food	2.32	(0.78)	2.34 (0.78)	2.29 (0.78)	0.122	2.33 (0.78)	2.27 (0.80)	0.229
Ultra-processed Food	2.31	(0.91)	2.36 (0.92)	2.24 (0.89)	0.008 **	2.31 (0.91)	2.29 (0.90)	0.544
Sugary drinks	1.43	(0.66)	1.44 (0.65)	1.42 (0.67)	0.307	1.43 (0.66)	1.39 (0.67)	0.192
Juice	1.23	(0.54)	1.23 (0.53)	1.25 (0.56)	0.513	1.23 (0.53)	1.27 (0.61)	0.489
Energy drinks	1.06	(0.30)	1.03 (0.20)	1.10 (0.39)	<0.001 **	1.06 (0.28)	1.09 (0.38)	0.267
Coffee	1.76	(0.71)	1.72 (0.69)	1.81 (0.72)	0.013 **	1.75 (0.70)	1.82 (0.74)	0.198

* Mann–Whitney-U test; ** Statistically significant.

## Data Availability

Data presented in this study are available upon reasonable request to the corresponding author.

## Appendix A

**Table A1:** Eigenvalues of the PCA between sport and lifestyle and health variables (Components and percentage of variance explained).

Component	Eigenvalue	% of Variance	Cumulative %
1	2.692	22.43	22.4
2	1.890	15.75	38.2
3	1.556	12.97	51.2
4	1.146	9.55	60.7
5	0.990	8.25	68.9
6	0.881	7.34	76.3
7	0.791	6.59	82.9
8	0.699	5.82	88.7
9	0.510	4.25	93.0
10	0.373	3.11	96.1
11	0.245	2.04	98.1
12	0.228	1.90	100

**Table A2:** Eigenvalues of the PCA between sport and nutrition variables (components and percentage of variance explained).

Component	Eigenvalue	% of Variance	Cumulative %
1	2.410	20.08	20.1
2	1.845	15.37	35.5
3	1.264	10.53	46.0
4	1.032	8.60	54.6
5	0.980	8.16	62.8
6	0.882	7.35	70.1
7	0.773	6.44	76.5
8	0.708	5.90	82.4
9	0.676	5.63	88.1
10	0.650	5.42	93.5
11	0.538	4.48	98.0
12	0.243	2.03	100

## Appendix B

**Table A3:** Comparison between physical activity habits and socio-demographic variables.

		Mean (SD)	Mean (SD)	Mean (SD)	
Education		Low Education	High Education		*p*-value *
	At work or in the daily commute				
	Days Intense physical activity at work	1.46 (1.72)	0.76 (1.34)		<0.001 **
	Minutes Intense physical activity at work	63.3 (235)	33.3 (160)		<0.001 **
	Days Moderate physical activity at work	1.68 (1.81)	1.08 (1.60)		<0.001 **
	Minutes Moderate physical activity at work	65.6 (168)	37.9 (113)		<0.001 **
	Days a week Walking or cycling	4.02 (2.60)	4.25 (2.52)		0.087
	Minutes a week Walking or cycling	54.5 (114)	38.1 (52.1)		0.032
	In leisure time				
	Days a week High-intensity sport	2.39 (2.13)	2.43 (2.05)		0.625
	Minutes a week High-intensity sport	51.2 (61.8)	20.5 (51.3)		0.248
	Days a week Moderate-intensity sport	2.60 (2.08)	2.66 (2.05)		0.603
	Minutes a week Moderate-intensity sport	67.5 (281)	69.9 (290)		0.302
	Daily lying time (minutes)	331 (3956)	238 (1556)		0.557
	Total minutes at work or in the daily commute plus minutes in leisure time				
	Total minutes a week High-intensity sport	115 (248)	83.9 (172)		<0.001 **
	Total Minutes a week Moderate-intensity sport	133 (368)	108 (313)		0.002 **
Incomes		Low Income	Medium-High Income		*p*-value *
	At work or in the daily commute				
	Days Intense physical activity at work	1.36 (1.69)	0.87 (1.43)		<0.001 **
	Minutes Intense physical activity at work	58.3 (251)	39.8 (167)		<0.001 **
	Days Moderate physical activity at work	1.60 (1.76)	1.17 (1.68)		<0.001 **
	Minutes Moderate physical activity at work	61.8 (179)	42.3 (114)		0.003 **
	Days a week Walking or cycling	4.17 (2.55)	4.08 (2.57)		0.242
	Minutes a week Walking or cycling	49.5 (106)	42.0 (69.8)		0.089
	In leisure time				
	Days a week High-intensity sport	2.32 (2.09)	2.48 (2.07)		0.391
	Minutes a week High-intensity sport	49.3 (60.7)	52.7 (53.8)		0.075
	Days a week Moderate-intensity sport	2.71 (2.12)	2.62 (2.05)		0.499
	Minutes a week Moderate-intensity sport	75.7 (344)	68.9 (272)		0.383
	Daily lying time (minutes)	350 (4236)	197 (1034)		0.044
	Total minutes at work or in the daily commute plus minutes in leisure time				
	Total minutes a week High-intensity sport	108 (262)	92.5 (181)		0.485
	Total Minutes a week Moderate-intensity sport	137 (427)	111 (298)		0.491
Family living		Living with family	Living without family		*p*-value *
	At work or in the daily commute				
	Days Intense physical activity at work	1.10 (1.63)	1.04 (1.53)		0.888
	Minutes Intense physical activity at work	46.7 (168)	45.7 (202)		0.368
	Days Moderate physical activity at work	1.36 (1.80)	1.32 (1.69)		0.800
	Minutes Moderate physical activity at work	57.4 (178)	47.4 (127)		0.533
	Days a week Walking or cycling	4.80 (2.31)	3.98 (2.59)		<0.001 **
	Minutes a week Walking or cycling	57.9 (92.1)	41.5 (81.3)		<0.001 **
	In leisure time				
	Days a week High-intensity sport	2.72 (2.06)	2.33 (2.08)		0.002 **
	Minutes a week High-intensity sport	58.0 (67.8)	48.9 (52.1)		0.074
	Days a week Moderate-intensity sport	2.83 (2.07)	2.58 (2.06)		0.062
	Minutes a week Moderate-intensity sport	57.6 (57.4)	71.9 (320)		0.107
	Daily lying time (minutes)	516 (5576)	213 (1337)		0.175
	Total minutes at work or in the daily commute plus minutes in leisure time				
	Total minutes a week High-intensity sport	105 (194)	94.6 (211)		0.555
	Total Minutes a week Moderate-intensity sport	115 (197)	119 (366)		0.494
Living situation		Living alone	Living with others		*p*-value ^*^
	At work or in the daily commute				
	Days Intense physical activity at work	1.04 (1.56)	1.06 (1.55)		0.702
	Minutes Intense physical activity at work	42.4 (190)	46.3 (196)		0.284
	Days Moderate physical activity at work	1.49 (1.87)	1.31 (1.70)		0.436
	Minutes Moderate physical activity at work	61.7 (205)	48.0 (128)		0.829
	Days a week Walking or cycling	4.48 (2.46)	4.11 (2.56)		0.080
	Minutes a week Walking or cycling	50.0 (80.2)	44.3 (84.4)		0.236
	In leisure time				
	Days a week High-intensity sport	2.71 (2.12)	2.38 (2.07)		0.043
	Minutes a week High-intensity sport	55.3 (67.8)	50.3 (54.2)		0.606
	Days a week Moderate-intensity sport	2.97 (2.18)	2.59 (2.04)		0.045
	Minutes a week Moderate-intensity sport	57.8 (61.9)	70.3 (303)		0.489
	Daily lying time (minutes)	740 (7643)	218 (1277)		0.098
	Total minutes at work or in the daily commute plus minutes in leisure time				
	Total minutes a week High-intensity sport	97.7 (2520)	96.6 (5000)		0.454
	Total Minutes a week Moderate-intensity sport	120 (227)	118 (349)		0.706

* Mann–Whitney-U test; ** Statistically significant.

Table A4 shows the differences in the practice of physical activity according to the type of diet.

**Table A4:** Population type of diet.

Special Diet	N	%
Mediterranean	1312	85.5
Vegan	0	0
All kinds of vegetarian	62	4.0
Intermittent fasting	44	2.9
Others	116	7.6

Table A5 shows the differences in the practice of physical activity according to the type of diet. Significant differences were observed in the number of days per week participants engaged in walking or cycling (*p* = 0.024) and high-intensity sports (*p* = 0.010). Daily lying time also showed a significant difference (*p* = 0.004), particularly between those following a Mediterranean diet and other types of diets (*p* = 0.011).

**Table A5:** Differences in the practice of physical activity according to the type of diet.

Variables	*p*-Value ^$^	
At work or in the daily commute		
Days Intense physical activity at work	0.194	
Minutes Intense physical activity at work	0.296	
Days Moderate physical activity at work	0.256	
Minutes Moderate physical activity at work	0.560	
Days a week Walking or cycling	0.024 **	Vegetarian vs. Others: 0.017
Minutes a week Walking or cycling	0.862	
In leisure time		
Days a week High-intensity sport	0.010 **	Mediterranean vs. Vegetarian: 0.039
Minutes a week High-intensity sport	0.539	
Days a week Moderate-intensity sport	0.031	
Minutes a week Moderate-intensity sport	0.373	
Daily lying time (minutes)	0.004 **	Mediterranean vs. Others: 0.011
Total minutes at work or in the daily commute plus minutes in leisure time		
Total minutes a week High-intensity sport	0.482	
Total Minutes a week Moderate-intensity sport	0.296	

^$^ Kruskal Wallis test, (** significative differences *p* < 0.05).
